# Identifying Quality Indicators Used by Patients to Choose Secondary Health Care Providers: A Mixed Methods Approach

**DOI:** 10.2196/mhealth.3808

**Published:** 2015-06-05

**Authors:** Dominic King, Sameer Zaman, Saman Sara Zaman, Gurnaaz Kaur Kahlon, Aditi Naik, Amar Singh Jessel, Niraj Nanavati, Akash Shah, Benita Cox, Ara Darzi

**Affiliations:** ^1^ Imperial College London London United Kingdom; ^2^ Imperial College London South Kensington Campus London United Kingdom

**Keywords:** mHealth, patient choice, mobile phone, hospital ratings

## Abstract

**Background:**

Patients in health systems across the world can now choose between different health care providers. Patients are increasingly using websites and apps to compare the quality of health care services available in order to make a choice of provider. In keeping with many patient-facing platforms, most services currently providing comparative information on different providers do not take account of end-user requirements or the available evidence base.

**Objective:**

To investigate what factors were considered most important when choosing nonemergency secondary health care providers in the United Kingdom with the purpose of translating these insights into a ratings platform delivered through a consumer mHealth app.

**Methods:**

A mixed methods approach was used to identify key indicators incorporating a literature review to identify and categorize existing quality indicators, a questionnaire survey to formulate a ranked list of performance indicators, and focus groups to explore rationales behind the rankings. Findings from qualitative and quantitative methodologies were mapped onto each other under the four categories identified by the literature review.

**Results:**

Quality indicators were divided into four categories. Hospital access was the least important category. The mean differences between the other three categories hospital statistics, hospital staff, and hospital facilities, were not statistically significant. Staff competence was the most important indicator in the hospital staff category; cleanliness and up-to-date facilities were equally important in hospital facilities; ease of travel to the hospital was found to be most important in hospital access. All quality indicators within the hospital statistics category were equally important. Focus groups elaborated that users find it difficult to judge staff competence despite its importance.

**Conclusions:**

A mixed methods approach is presented, which supported a patient-centered development and evaluation of a hospital ratings mobile app. Where possible, mHealth developers should use systematic research methods in order to more closely meet the needs of the end user and add credibility to their platform.

## Introduction

### Background

Patient choice has come to prominence in the United Kingdom with the advent of the National Health Service (NHS) Choose and Book and representation in key health policies such as Choice Matters [1] and High Quality Care for All: NHS Next Stage Review [2]. Patients in many countries (including the United Kingdom, Netherlands, and the United States) can now vote with their feet and choose health providers that fit best with their preferences and needs [3]. Providing choice is thought to be important in promoting competition between providers, with the goal of improving both the quality and efficiency of care [4]. While the actual evidence supporting the contribution of patient choice to cost control and quality of care is mixed [5], improving patient choice and shared decision making remains a stated objective of different health systems worldwide.

Service users trying to select between different health providers can use information from a variety of sources. Increasingly, patients are using websites that provide information about the comparative quality of health care from different providers [6]. For example, patients can compare hospitals using a wide range of quality and performance indicators such as waiting times, staffing, and patient safety on the website of the Care Quality Commission, the independent regulator of health care in England [7]. Service users are also turning to information based on the experiences of other patients when making a choice of provider. While patients have long used the experiences of friends and family in choosing hospitals, service users can now more systematically access collected information on patient experience (eg, Consumer Assessment of Health care Providers and Systems reports in the United States [8] and NHS Choices in the United Kingdom [9]). There is evidence that information based on patient experience is considered at least as important by service users choosing between different providers as different performance indicators provided by hospitals or reporting bodies [10].

An interesting recent development is the advent of patient rating websites such as PatientOpinion [11] and IWantGreatCare [12] (United Kingdom) and Rate MDs [13] (United States and Canada), where patients express views about the care they have received in much the same way as they might rate a hotel on a travel website. Users rating and commenting on the health care they receive is only set to increase with growing access to the Internet (particularly through increasingly ubiquitous mobile phone and tablet devices) [14]. Our research team has previously shown that online discretionary patient ratings can be useful in providing reliable information about health care quality [15,16].

This paper describes the process that was undertaken in the development of a hospital ratings platform for a consumer health care app. The aim to incorporate the best available evidence lies in sharp contrast to the majority of health related apps [17]. By working with patients and members of the public, we also sought to meet end-user needs often overlooked [18]. To develop the hospital ratings service, research was undertaken to determine which factors were considered important to individuals when choosing a nonemergency health care provider. This paper describes how established research methods can be used in the development of new mHealth apps.

### Objectives

The aim of this study was to generate a list of quality indicators from the general public that were deemed important when choosing nonemergency secondary health care services along with the rationales for these choices, with the intention of using the findings in a new mHealth hospital ratings platform. Further, we aimed to illustrate the importance of rigorous research methodologies to underpin the development of mHealth technologies. The study was considered as part of a service evaluation, and ethics approval was not required. The study was conducted in London between November 2011 and June 2013.

## Methods

### Literature Review

As the first part of a mixed methods approach to identify which factors were considered most important to people when choosing nonemergency secondary health care providers in the United Kingdom, a review of existing literature (both academic and grey literature) was conducted. Publications were included only if they described patient choice in the *United Kingdom* health system so as to avoid confounding factors in the context of other health systems. For example, although there are many relevant articles from the United States, the differences between the largely privately funded United States and publicly funded United Kingdom systems may influence what users consider important when choosing a hospital. Patient choice did not feature prominently in national health policy until more recently [1,2]. A pilot literature review revealed a dearth of high quality research formally investigating patient choice in secondary health care prior to 2005. The patient choice agenda was first investigated when Lewis began studying patients’ attitudes towards choice of hospital in the context of waiting times for cardiothoracic surgery [19]. Studies were therefore excluded if they had been carried out prior to 2005.

### Survey

The aim of the questionnaire was to formulate a ranked list of quality indicators. The survey was completed by participants with a member of the research team at hand to explain any terms or answer questions. Care was taken to ensure that facilitators did not directly ask questions to avoid leading or influencing participants choices. A power calculation [20] for a study comparing the attitudes regarding choosing secondary health care between two groups (general public and out-patients attending clinic appointments at the hospital) determined a target total sample size of 400 (population of London, 2001: 7,172,091 [21]; standard of error 0.05), thus two groups of approximately 200 participants. Members of the general public were recruited (n=201), and the data are presented here. Data collected from the second group (patients at the hospital) are beyond the scope of this manuscript. An initial pilot questionnaire was conducted on 20 individuals prior to its wider use to identify and correct any unforeseen problems. Quota sampling was used to estimate size of target groups to ensure accurate representation of ages and genders [22]. Four age categories were formed by combining existing categories from 2001 United Kingdom national census data [21]; target proportions for each age category were based on urban population proportions from the same census. Due to time and resource constraints, convenience sampling was then used to collect data, with checks to ensure collected data approximated the census age proportions. A higher proportion of people aged 18 to 35 years were included compared to people 60 years and older to account for lower usage of mHealth apps in the latter age group as described by previous investigators [23].

Inclusion criteria were English-speaking adults (18 years and older) UK residents. In order to ensure data collection was feasible under time and resource constraints, convenience sampling was then employed to recruit participants in six separate locations in central and greater London to provide greater geographical spread and wider generalizability of the data. Questionnaire collection ceased once the number of participants in each demographic group approached the estimated targets.

Informed consent was obtained for each participant completing the questionnaire. Participants were asked to provide demographic information and rank a predetermined list of quality indicators in order of importance. An ordinal scale was used, where respondents were asked to rank the factors in order of importance, first within their categories and then the categories themselves. This allowed us to assess the *relative* importance of the factors, as opposed to *absolute* importance [24]. Data were collated using Excel (Microsoft Corporation); SPSS (IBM Corporation) was used to undertake statistical analysis. Differences in mean ranks within categories were determined using the Friedman test, with *P*<.05 considered to be statistically significant.

### Focus Groups

Focus groups were used to discuss the rationales behind the quality indicators considered to be important. Convenience sampling was used to recruit participants due to time and cost constraints, and referrals from initial recruits were used for further recruiting. We sought to recruit an equal representation of genders and ages in order to increase the generalizability of the results. Due to resource and time constraints it was not possible to match the age stratification of focus group participants with questionnaire respondents. The median age of participants at pilot focus groups was 40 years; therefore, participants were stratified by age and gender using this as a marker of division (males under 40 years, females under 40 years, males over 40 years, females over 40 years) to enable timely data collection and efficient analyses. Four focus groups were conducted, each comprising 6 individuals, with a gradual shift from broad open questions to narrow, focused questions [25]. Written consent was gained from each participant in advance. A final script of questions for the focus groups was confirmed following a restructuring of a preliminary script that had been piloted. Each focus group lasted for approximately 90 minutes and was led by one researcher acting as an impartial facilitator and one as an assistant moderator. All recordings were transcribed verbatim, and thematic analysis [26] was used to identify common themes.

## Results

### Characteristics of Quality Indicators

#### Literature Review

The full findings of the literature review are beyond the scope of this manuscript but we include key details pertinent to subsequent survey and focus group development. Searches of the grey literature were included due to a paucity of peer-reviewed publications. Five publications were identified for critical review [24,27-30]. Regular surveys commissioned by the UK Department of Health regarding the subject of choice in health care were also examined. A summary of included literature is presented in Textbox 1. From these, a list of choice factors important to patients selecting a health provider was devised. The factors identified in the literature review were separated into four categories of quality indicators: hospital statistics, hospital staff, hospital facilities, and hospital access (Textbox 2). These categories formed the questionnaire and informed the discussion topics for focus groups.

**Summary of the literature review: key factors guiding patient choice.**

Understanding Patients’ Choices at the Point of Referral [28]

Areas of investigation

- Factors influencing patients when choosing hospitals
- Developing an algorithm to predict demand for particular services

Key findings

- Views provider quality as extremely important: 80%
- Values low mortality rates, infection rates, and readmission rates: 90%
- Views waiting times as important: 55%
- Views primary care provider influence as important: 60% (most important factor: 2%)
- Views travel as important: 30% (most important factor: 15%)
- Preference for lower travel costs was observed

Patient Choice: How Patients Choose and Providers Respond [24]

Areas of investigation

- Patient considerations when choosing health care
- Primary care provider response to the notion of patient choice and subsequent support of patient choice

Key findings

- Considers personal experience: 41%
- Judges primary care provider advice as important: 36%
- Factors identified in order of importance (graded out of 3):
  - Cleanliness (2.6)
  - Quality of care (2.5)
  - Standard of facilities (2.1)
  - Friendliness (2.1)
  - Waiting time (2.1)
  - Experience (2.0)
  - Proximity (2.0)
  - Waiting room (1.8)
  - Convenience of appointment time (1.8)
  - Consultant of choice (1.7)
  - Fixtures and fittings (1.5)
  - Accessibility (1.2)
  - Food (1.2)
  - Travel Cost (1.0)

Report on the National Patient Choice Survey [30]

Area of investigation

- The single most important factor patients consider when choosing a secondary health care provider

Key findings

- Rates proximity to home/work as single most important factor: 38%
- Factors reported as being most important:
  - Previous experience of the hospital: 12%
  - Waiting times: 10%
  - Previous good experience: 6%
  - Quality of care: 5%
  - Accessibility: 5%

Choosing a High Quality Hospital: The Role of Nudges, Scorecard Design, and Information [29]

Areas of investigation

- Information important to patients when choosing a hospital
- How presentation of information affects decisions

Key findings

- Values information relevant to the patient (eg, their consultant, condition)
- Format of information plays a role in its interpretation (ie, only patients with high levels of numeracy can interpret mortality ratios)
- Factors deemed important: waiting times, MRSA rates, quality of service, doctors’ expertise, cleanliness, distance, being treated with respect

London Patient Choice Project Evaluation: A Model of Patients’ Choices of Hospital from Stated and Revealed Preference Choice Data. [27]

Areas of investigation

- Factors used by patients when deciding to accept alternative treatment
- Weighing the relevant factors
- Trade-offs patients make when considering different factors

Key finding

- Less likely to take up offer of quicker treatment elsewhere if the alternative hospital has a worse reputation or the appointment involves increased travel time, results in patient paying for transport or requires nonlocal follow-up care.

**Quality indicators identified by the literature review.**

Hospital statistics [24,28-30]

- MRSA infection rates
- Readmission rates
- Mortality rates
- Wound infection rates
- Waiting times

Hospital staff [24,29]

- Friendliness
- Respectfulness
- Competence

Hospital facilities [24,29]

- Cleanliness
- Hygiene
- Availability of single-sex wards
- Quality of food
- Standard of facilities

Hospital access [24,27-30]

- Distance from home
- Cost of travel
- Time to travel
- Car parking availability

#### Questionnaire

**Figure 1:**
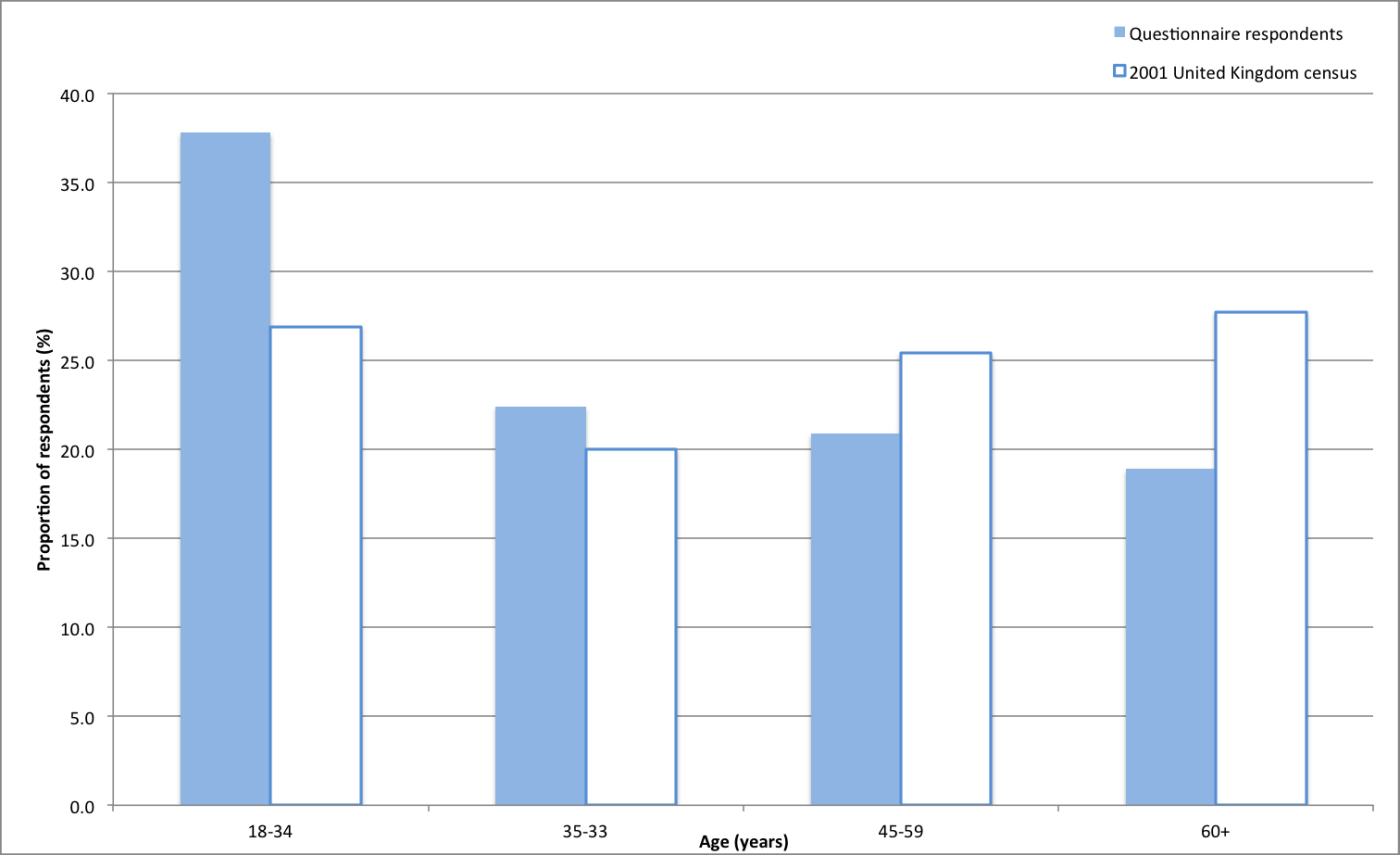
Age spread of questionnaire respondents compared with 2001 United Kingdom census respondents.

Members of the general public completed the questionnaire (93 male, 108 female, n=201). The age spread of the sample compared to 2001 population proportions can be seen in Figure 1. Respondents ranked quality indicators in order of importance within their specified categories (ie, 1 through 5 with 1 being the most important). Based on mean rankings, the quality indicators were arranged in order of preference within respective categories (Table 1). Similarly respondents were asked to rank the overall categories (statistics, staff, facilities, and access) (Table 2). The Friedman test was used to determine the statistical significance of the differences between the mean ranks obtained for the quality indicators within and between categories to determine the true order (Multimedia appendix 1). Final ranked order of quality indicators and categories was determined after statistical analyses (Table 3).

While three of the categories (statistics, staff, facilities) were deemed equally important, quality indicators under the category of access were considered to be of less importance. Within each group some indicators were seen as being more important than others. Regarding staff, competence was seen as being significantly more important than friendliness and respectfulness. In terms of facilities, up-to-date facilities and the cleanliness of the premises were seen as equally important but more so than the other factors. In the category of statistics, infection rates, mortality rates, complication rates, and waiting times were of equal importance; statistics regarding readmission rates were seen as less important. Regarding access, ease of travel was more important that the cost and availability of car parking.

**Table 1:** Mean rankings of quality indicators within each category.

Categories	Quality indicators	Mean rankings
Hospital statistics	Infection rates	2.2
	Mortality rates	2.8
	Waiting times	3.0
	Complication rates	3.2
	Readmission rates	3.8
Hospital staff	Competence	1.3
	Friendliness	2.3
	Respectfulness	2.3
Hospital facilities	Clean premises	1.8
	Up-to-date equipment	2.0
	Good food	4.1
	Disabled facilities	4.2
	Single sex wards	4.4
	Appealing appearance	4.5
Hospital access	Ease of travel	1.8
	Cost/availability of car parking	2.1

**Table 2:** Mean rankings between categories.

Overall groups	Mean rankings
Hospital facilities	2.2
Hospital staff	2.3
Hospital statistics	2.5
Hospital access	3.1

**Table 3:** Overall rankings of quality indicators within and between categories.

Categories		Ranking of quality indicators
**Hospital statistics**		
	More important	Infection rates, mortality rates, complication rates, waiting times
	Less important	Readmission rates
**Hospital staff**		
	More important	Competence
	Less important	Friendliness, respectfulness
**Hospital facilities**		
	More important	Clean premises, up-to-date equipment
	Less important	Good food, disabled facilities, single sex wards, appealing appearance
**Hospital access**		
	More important	Ease of travel
	Less important	Cost/availability of car parking
**Overall categories**		
	More important	Hospital statistics, staff, facilities
	Less important	Hospital access

#### Focus Groups

**Figure 2:**
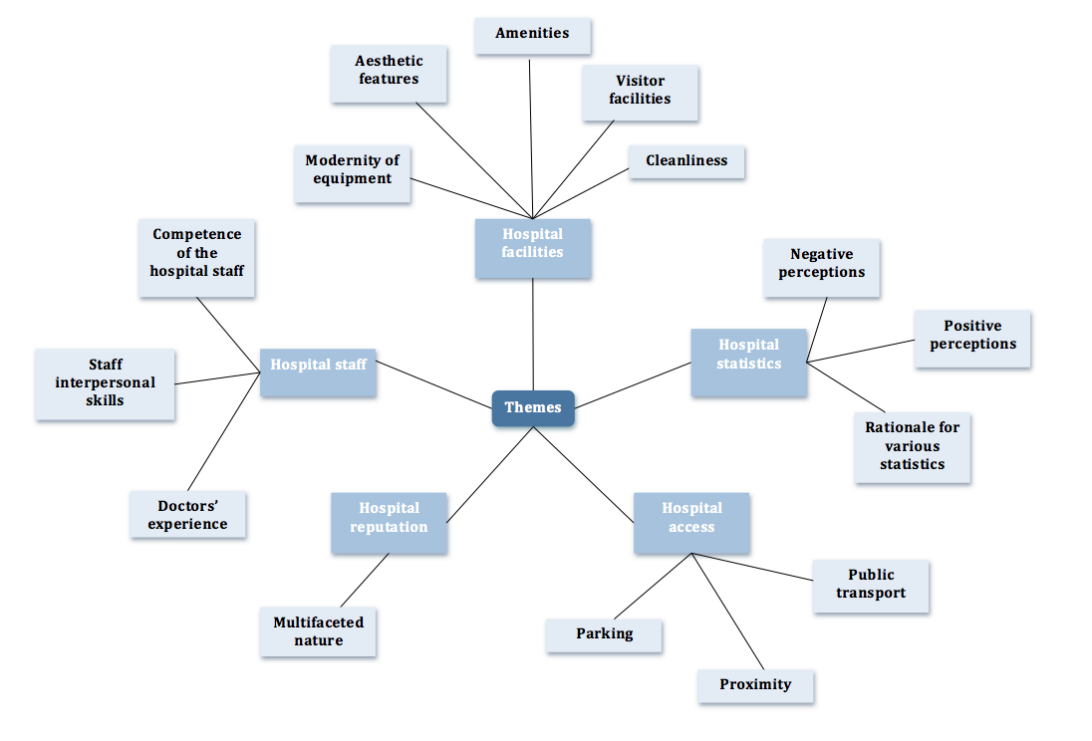
Thematic map of qualitative data from focus groups.

Four focus groups were used to explore the rationales behind rankings formulated from the questionnaire. Thematic analysis was conducted by performing manual coding [26], from which a collective list of codes was assembled. Overarching subthemes, and subsequently themes, were identified and reviewed. The themes, subthemes, and codes for different preferences established during the analysis are presented in Table 4 and are visually represented in Figure 2. The findings from both the quantitative and qualitative methodologies were mapped to each other under the four categories identified by the systematic literature review.

**Table 4:** Themes, subthemes, and codes from focus groups.

Theme	Subtheme	Codes
Hospital reputation	Multifaceted nature of reputation	Important because it encompasses everything
		Important because it reflects the facilities at the hospital
		Important because it reflects the competence of the hospital staff
Hospital statistics	Rationale for choosing various statistics	Infection rates are important because they are frequently reported to the media
		Waiting times are important because being treated quickly is my main concern
		Waiting times are important because they reflect the hospital’s efficiency
		Departmental statistics are more relevant because they are specific to the situation
		Waiting times are important because I do not want to spend too much time at the hospital
		MRSA rates are important because of the risks faced by visitors
		Mortality rates may not be the best indicators because better hospitals may undertake more challenging cases
	Negative perceptions of statistical descriptors	Not relevant in the context of routine procedures
		Not important because they can be manipulated
		Not important because they are negatively exaggerated in the media
	Positive perceptions of statistical descriptors	Important because they are true facts about hospital quality
Hospital staff	Competence of hospital staff	Seeing specialists is important because they are more skilled
		Competence is the most important because my main aim is being treated properly
		Important because I want to be treated correctly, regardless of friendliness
		Important because it reflects staff experience
		Most important because I would travel further to ensure it
		Important because it encompasses interpersonal skills too
	Staff interpersonal skills	Important because I expect to be treated fairly
		Important for nurses because they are responsible for making you comfortable
		Important because I feel more reassured with doctors and nurses that I know
		Important because they have an impact on recovery rates
	Doctors’ experience	Younger doctors are not good because they are inexperienced
		Qualifications are important because they reflect competence
Hospital facilities	Modernity of equipment and cleanliness of hospital	Important for outpatients because there is only a limited time to experience it
		Not important because it is assumed to be equally up-to-date at all hospitals
		Cleanliness of the hospital is the most important factor for outpatients because they are only there for a short time
	Aesthetic features and amenities of patient comfort	Important because it reflects the comfort of the hospital
		Not important because it is assumed that all hospitals are equally clean
		Important because poor aesthetics can lead to depression
		Not important because they do not affect health care
		Not important so long as staff is competent
		Important because I would like to see the hospital before choosing to be treated there
		TV facilities are important because one might be staying at the hospital for an extended time
		Facilities are not important because they are subject to individual experience
	Facilities for visitors	Food and drink facilities are important to ensure comfort for visitors
		Visiting times at the hospital should be flexible because the convenience of visitors is important
		Overnight facilities for visitors are important so they can spend longer time with the patient
Hospital access	Parking at and around the hospital	Parking charges are important because the may affect my visitors
		Not important because I do not have a car
		Availability is important because I drive
	Proximity of the hospital	Not important because I am willing to travel further if other factors are better satisfied
		More important so that visitors can visit me easily
		Important because I do not have a car
	Public transport	Important because parking at/around the hospital is too expensive

### Interpretation of Data

#### Hospital Statistics

Qualitative analyses of the focus groups showed that people consider a wide range of statistical descriptors. Participants reasoned that information regarding infection rates was an important consideration, mainly due to the extensive media coverage: this may account for why infection rates were ranked joint highest in this category during the quantitative analyses. Elsewhere, the qualitative data disagreed with the survey findings, asserting that readmission rates were also considered to be important by the focus group participants because people felt the rates reflected the success of a specific treatment or condition. Furthermore, unlike the survey respondents, the focus group participants did not feel that mortality rates were a valuable quality indicator because it was reasoned that a hospital may undertake more challenging cases, which could inflate mortality rates despite the hospital faring well on other indicators such as staff competence. Participants also speculated that in many cases departmental statistics may be more meaningful than those describing the hospital overall due to the variation between departments within a hospital, leading to potential misrepresentation of the overall hospital statistics. This interesting finding was not captured by the quantitative analyses and highlights a potential avenue for future research.

#### Hospital Staff

Staff competence was identified to be the most important factor by the quantitative analyses, followed by friendliness and respectfulness equally. Qualitative analyses were in agreement, with participants confirming the importance of receiving treatment by competent staff, with some even stating that they would travel further than their nearest hospital in order to secure treatment by a competent doctor. However, the focus groups identified that there is no single measure by which the public could judge competence. Rather competence was defined as a compound of experience, qualifications, place of education, or even the possession of excellent interpersonal skills.

#### Hospital Facilities

The quantitative analysis identifies cleanliness and modernity of equipment as the two highest ranked indicators. Rationales elicited from the focus groups shed light on why this may be the case. Members of the focus groups felt cleanliness to be very important in hospitals but did not necessarily seek out data about it when making a choice of hospital. It was suggested that this was a consequence of the assumption that cleanliness is the same in all hospitals. There was less consensus regarding the importance of modern equipment, although some certainly felt access to the latest technologies to be important.

#### Hospital Access

The quantitative analyses showed that this category was less important than the other three. Within this category, ease of traveling to the hospital was significantly more important than parking and cost of travel. Focus groups revealed that the proximity of the hospital to home or work was an important consideration. However, this is very much dependent on the severity of illness and the availability of treatment, with participants expressing that they may be willing to travel beyond their most proximal hospital in order to benefit from a higher quality of care. Therefore, it appears that the importance of this indicator may depend on the context of the decision.

## Discussion

### Key Findings and Recommendations

Service users across many health systems are now offered a wider choice of health care providers and increasingly have at their disposal a wide variety of factors to consider when making these decisions. The rapid adoption of mobile phones and tablet devices has enhanced access to information about different hospitals by making it possible for patients to view and share this information at any time and while on the move [31].

This study collates the existing literature regarding which factors are considered important for consumers in this context, contributes a categorized and ranked list of quality indicators, and reconciles the rationales underpinning these decisions. Furthermore, this study demonstrates how this information can be harnessed in the context of developing a robust user-generated ratings platform for use on mobile communication technologies.

Although mobile technologies are frequently put forward as a solution to challenges in health informatics, there is often a lack of rigorous research underpinning their development and evaluation. This project illustrates the importance of sound research methodology when developing these strategies by employing a mixed methods approach to reconfigure the ratings service based on factors that the public held to be important in choosing nonemergency health providers.

Findings included that staff competence was the most important factor within the hospital staff category, with participants asserting that they would travel further than their nearest hospital to secure treatment under a doctor they perceived to be more competent. However, the qualitative analyses revealed that there is no single measure by which competence could be judged; rather it was a compound of many factors including amount of experience, qualifications, place of education, and interpersonal skills.

Cleanliness and modernity of equipment stood out as the two most important hospital facilities. This is concurrent with previous reports that people consider information about cleanliness when researching a hospital [32,33]. Qualitative analyses discovered that while this was a factor deemed to be highly important, it was not widely sought after. Participants suggested this might be due to a commonly held assumption that hospitals are of equal cleanliness, therefore only those hospitals with a remarkably poor reputation for cleanliness may be of note. This was also the case for modernity of equipment. Future mHealth developers should reflect on this subtlety in order to include factors that are not only important but also highly sought after to avoid information overload for users.

**Figure 3:**
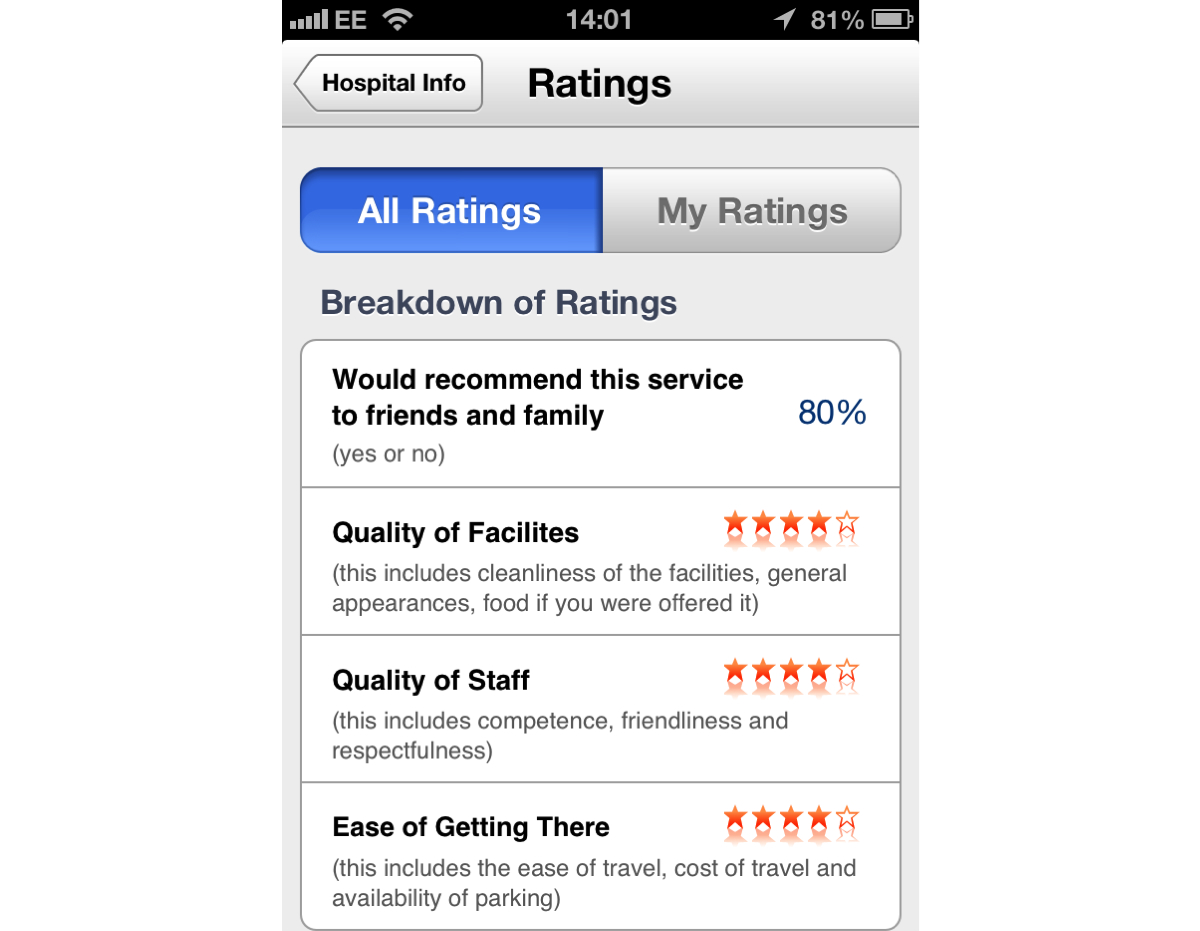
Screenshot of Wellnote ratings platform.

Participants could not differentiate level of importance between various types of hospital statistics. Hospital-wide statistics may be of limited use to users who would be more interested in department-specific statistics. Moreover, users appreciate that overall hospital statistics may not be an accurate representation of an their department of interest due to interdepartmental variation. Conversely it may be argued that an inability to compare the importance of statistical descriptors may reflect that they are poorly understood by users. This may explain equal significance attributed to individual statistics within this category and highlights the need for the careful inclusion of statistics that are relevant to the user’s individual health encounter in mHealth platforms (See Figure 3). Care must be taken by developers to ensure that presentation of statistics, including color coding or a glossary of terms, aids user interpretation. These subtleties may not be appreciated without formal research methods informing these strategies.

The fact that the categories of hospital staff, hospital facilities and hospital statistics were deemed equally important illustrates that users’ demands for information about hospitals are extensive and varied. mHealth developers should aim to provide information about these categories equally in order to reflect and satisfy these demands. Adequate provision of these varied factors requires an equally varied presentation of information. For example, participants asserted that graphs and percentages provided objective evidence of statistical measures, whereas past users’ reviews were more useful in capturing complex domains such as staff competence. Therefore we recommend that mHealth developers include a range of formats as this study illustrates that each caters to different, and equally important, categories of quality indicators.

### Limitations

An important limitation of the study is that the questions were asked outside the context of mobile phones and mHealth. This was a purposeful decision as it was felt that doing so may lead to reduced applicability of this research. Further research is required specifically investigating whether the information consumers want in the context of an mHealth app is any different from the factors that are important when choosing secondary health care in general. The original study was adequately powered for a comparison between patient groups and the general public; the analyses included here may therefore by underpowered due to resource constraints. This study was unable to match age stratification between quantitative and qualitative stages. We recommend that future investigators attempt to do so to allow closer mapping of the two datasets.

### Conclusion

The huge interest in developing apps for mobile phone and tablet platforms to enhance health outcomes and service delivery—widely termed mHealth—has led to an “enthusiastic proliferation of untested methods” [17]. An evidence base needs to be developed to make this field credible and address the needs of the end-user. More attention needs to be paid to structuring app development in theory or best practice [34].

This study used a mixed methods approach to find that information about hospital staff, hospital facilities and hospital statistics are equally important to people when choosing a hospital. Information about getting to the hospital is least important. Staff competence is most important regarding hospital staff, which is a multifactorial domain best captured by past users’ reviews; cleanliness and modernity of equipment are most important regarding hospital facilities but are not actively sought after. People find it difficult to compare relative importance between various hospital statistics. Barriers to understanding statistics may be removed by use of graphs and percentages.

Users of health care demand a wide and varied range of information about hospitals. mHealth developers must determine which information is most relevant to their users’ needs and provide this in an accessible format. Less important information must be identified and removed to avoid information overload. A sophisticated appreciation of the complex needs of mHealth users is possible when these strategies are underpinned by rigorous research methods. This study demonstrates how a mixed methods approach can enhance mHealth solutions.

## Appendix

Multimedia Appendix 1 Tabulated *P* values of statistical comparison of mean rankings of quality indicators within and between categories.
