# Supplementary figures and images for: Identifying Quality Indicators Used by Patients to Choose Secondary Health Care Providers: A Mixed Methods Approach
Source: JMIR Mhealth Uhealth. 2015 Jun 5;3(2):e65. doi: 10.2196/mhealth.3808 (PMC4526909; doi:10.2196/mhealth.3808)

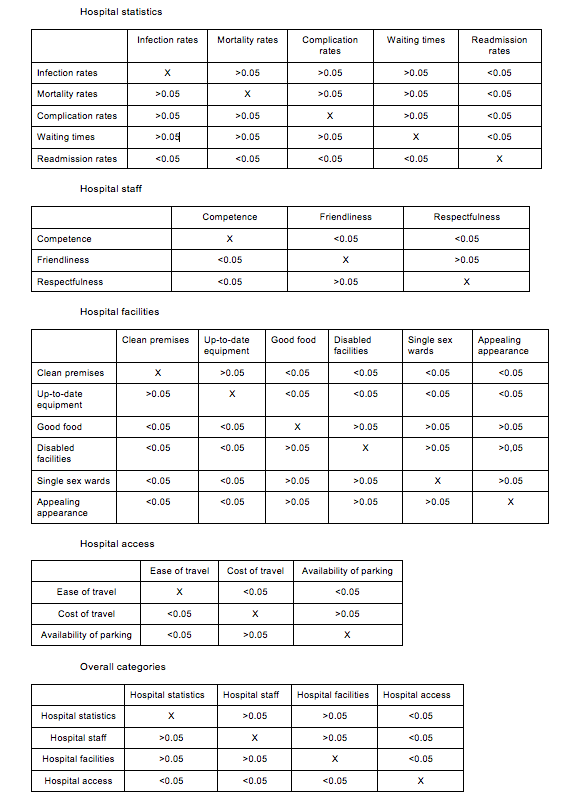

Supplement: Multimedia Appendix 1 [file mhealth_v3i2e65_app1.png]
